# EASIX as a predictor of 3-year mortality in aortic stenosis patients undergoing TAVR

**DOI:** 10.1007/s00392-025-02715-3

**Published:** 2025-07-23

**Authors:** Mustafa Mousa Basha, Baravan Al-Kassou, Christopher Gestrich, Marcel Weber, Thomas Beiert, Sebastian Zimmer, Farhad Bakhtiary, Georg Nickenig, Jasmin Shamekhi

**Affiliations:** 1 Heart Center, Department of Medicine II, University Hospital Bonn, Bonn, Germany; 2 Heart Center Bonn, Department of Cardiac Surgery, University Hospital Bonn, Bonn, Germany

**Keywords:** Aortic valve stenosis, Transcatheter aortic valve replacement, EASIX, Endothelial dysfunction

## Abstract

**Background:**

Endothelial dysfunction plays a crucial role in the progression of aortic stenosis (AS), and the Endothelial Activation and Stress Index (EASIX) has been proposed as a biomarker for predicting mortality in various clinical settings.

**Aims:**

Evaluating the predictive value of the EASIX for 3-year all-cause mortality in patients undergoing transcatheter aortic valve replacement (TAVR).

**Methods:**

We conducted a retrospective analysis of 1084 patients with severe AS, who underwent TAVR between 2013 and 2021 at the Heart Center Bonn. The EASIX was measured pre-procedural. The optimal cut-off (EASIX ≥ 1.65) was determined using the Youden index. Its association with 3-year mortality was assessed using Kaplan–Meier survival analysis and Cox regression models. The primary endpoint was 3-year all-cause mortality.

**Results:**

Patients with an EASIX ≥ 1.65 had significantly higher 3-year mortality compared to those with lower EASIX (45.8% vs. 27.7%, *p* < 0.001). In multivariate analysis, EASIX remained an independent predictor of mortality (HR = 1.4, 95% CI: 1.1–1.8, *p* = 0.010). ROC analysis revealed an area under the curve (AUC) of 63.0% for the EASIX; its predictive ability was inferior to the well-established cardiac biomarkers such as NT-proBNP (AUC = 70.2%) and troponin T (AUC = 69.8%).

**Conclusion:**

The EASIX is a significant predictor of 3-year all-cause mortality in patients undergoing TAVR. However, its predictive performance is lower than NT-proBNP and troponin T. Integrating EASIX with traditional cardiac biomarkers may enhance risk stratification in TAVR patients and improve personalized care.

**Graphical Abstract:**

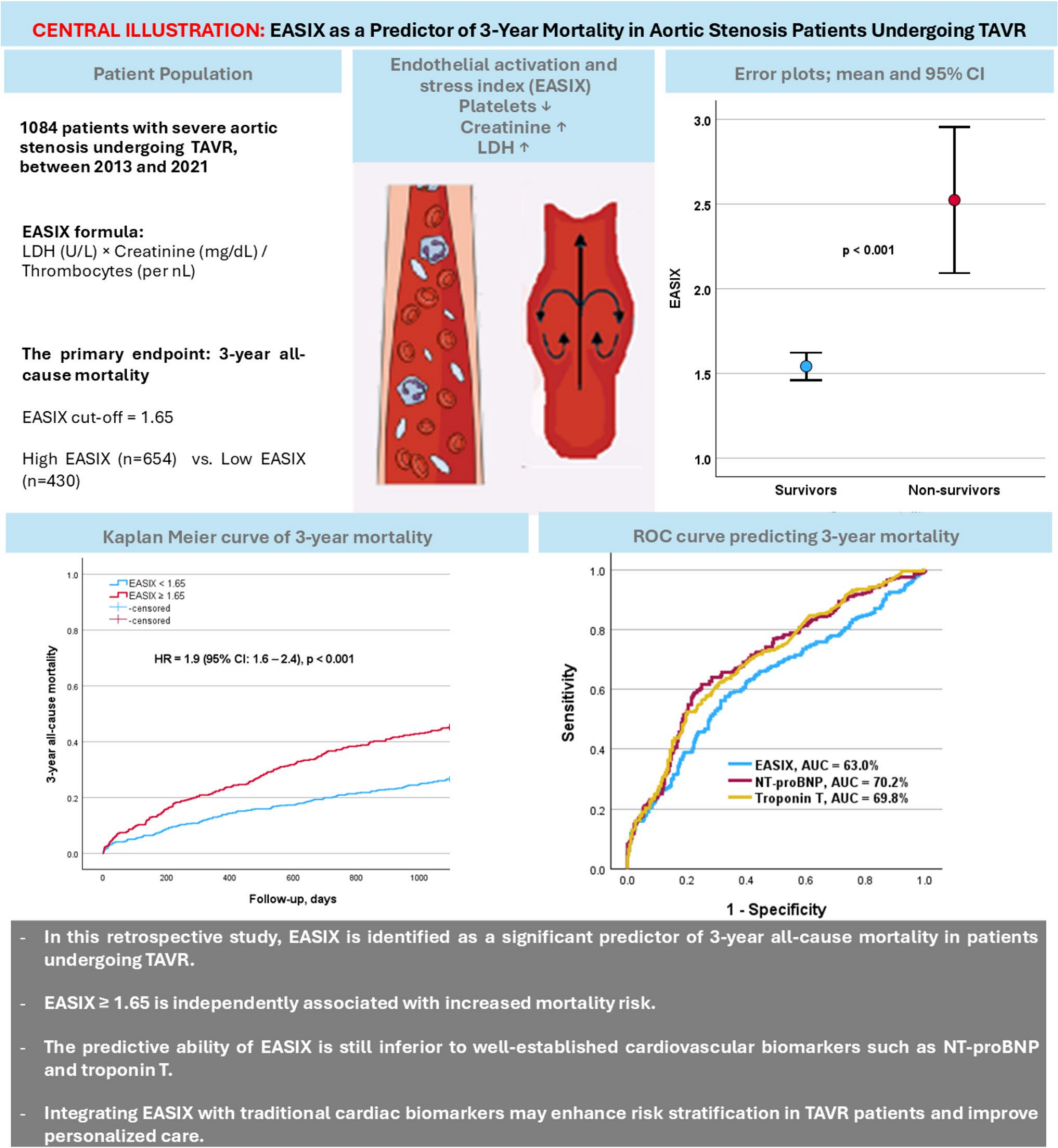

## Introduction

Aortic stenosis (AS) is the most common acquired heart valve disease and a socioeconomically challenging condition in the Western world [1]. Flow disturbances, per se, can critically impact endothelial function, as AS is also associated with cardiovascular diseases that are well-known to be accompanied by endothelial dysfunction [2]. Over the last decade, transcatheter aortic valve implantation (TAVR) has emerged as a transformative therapy for most patients with severe symptomatic AS [3]. Previous studies have demonstrated that endothelial cell-derived microvesicles decrease following TAVR [4]. Cardiac microvascular dysfunction [5] is associated with poor outcomes in patients with coronary artery disease (CAD) and endothelial complications are strongly associated with mortality in hematological patients [6].

The need to predict endothelial complications, particularly after allogeneic stem cell transplantation (alloSCT), led to the development of the EASIX (Endothelial Activation and Stress Index). The EASIX incorporates three relevant routine laboratory markers required to diagnose transplant-associated thrombotic microangiopathy (TAM) [7]. The EASIX formula integrates these changes into a single continuous value representing the three markers. The index enables prediction of endothelial complications, such as TAM after alloSCT, as early as before initiating chemotherapy for alloSCT [8].

In recent years, the EASIX has been established and validated as a biomarker to predict endothelial dysfunction and survival in patients after alloSCT and graft-versus-host disease (GVHD) [9]. Furthermore, the EASIX has demonstrated utility in predicting mortality due to sepsis after ICU admission [10], mortality in severe liver diseases [11], and mortality or severe disease progression in COVID-19 patients upon hospital admission [12]. Recently, an association between the EASIX and mortality in CAD patients and after acute myocardial infarction has also been described [13, 14].

The strong association of the EASIX with mortality across diverse contexts suggests that the EASIX may serve as a global marker of endothelial cell dysfunction. We hypothesize that the EASIX may also be a valuable predictor of outcomes in TAVR patients.

## Methods

### Patient population

We retrospectively analyzed the data of 1084 patients with symptomatic severe aortic stenosis and increased surgical risk who underwent TAVR between 2013 and 2021 at the Heart Center Bonn. In all patients, we had sufficient laboratory parameters to assess the EASIX. The retrospective use of patients’ data for scientific needs was approved by the local ethics committee, and written informed consent was obtained from all patients prior to the procedure.

Before the TAVR procedure, all patients underwent a careful evaluation including pre-interventional transthoracic and transesophageal echocardiography (including three-dimensional measurements) and had an interdisciplinary discussion with the local, institutional Heart Team. Coronary angiography was routinely performed in all patients as part of the preprocedural assessment. Details about patient screening, procedural techniques, and adjunctive medication have been described elsewhere [15].

### EASIX

The Endothelial Activation and Stress Index (EASIX) is calculated using the following formula: LDH (U/L) × Creatinine (mg/dL)/Thrombocytes (per nL) as described previously [16]. LDH, creatinine, and platelet counts were obtained 1 to 3 days prior to the intervention and routinely measured at the central laboratory of University Hospital Bonn. The EASIX parameters, along with the other laboratory values (e.g., Troponin T, NT-proBNP), were obtained from the same blood draw. Given the absence of established cut-off values for EASIX in patients with aortic valve stenosis, we utilized the Youden-index to define an optimal cut-off (EASIX = 1.65) to separate the overall population into a low and high EASIX group for our statistical analysis.

### Study endpoints

The primary endpoint of our study was the 3-year all-cause mortality. Follow-up was performed either by scheduled ambulatory visits or telephone interviews. Therefore, detailed information regarding the cause of death was not available.

### Statistical analysis

Normally distributed data are shown as mean ± standard deviation or as counts with percentages; non-normally distributed data is presented as a median with range or inter-quartile range (IQR). *p* values have been used for comparison using Student’s *t*-test, Fisher exact tests, or chi-squared tests as appropriate. Clinical event rates for outcomes are shown with the use of Kaplan–Meier curves and compared with the log-rank test. Individual risk factors for 3-year mortality were evaluated using logistic regression, estimating hazard ratios (HRs) and corresponding 95% confidence intervals. The sensitivity and specificity of the different risk scores were assessed via receiver operating characteristics (ROC) analysis. The degree of separability was determined through AUC (area under the curve) assessment. Youden index was calculated to define an optimal cut-off to separate the overall population into a low and high EASIX group.

Differences were considered statistically significant when *p* < 0.05. Statistical analyses were conducted with SPSS Statistics version 29.0 (IBM Corporation, Somers, NY, USA). The forest plot was created using JASP (Version 0.19.2). The investigators initiated the study, had full access to the data, and wrote the manuscript. All authors vouch for the data and its analysis.

## Results

A total of 1084 patients were included in the present analysis. The patient cohort presented with a mean age of 82.1 ± 6.1 years and an intermediate operative risk (EuroSCORE II: 5.5 ± 5.4, STS-Score: 5.8 ± 3.5). All patients underwent transfemoral TAVR. Follow-up outcome data were collected for all patients, with a mean follow-up duration of 841 ± 451 days (median: 1095 days; IQR: (627–1095) days).

### Baseline and procedural characteristics

Baseline characteristics according to the EASIX cutoff are presented in Table 1. Female patients were significantly more prevalent in the lower EASIX group (50.8% vs. 36.0%; *p* < 0.001), whereas age and BMI showed no significant differences between the groups (*p* = 0.497 and *p* = 0.359, respectively). Similarly, no significant differences were noted for the prevalence of chronic obstructive pulmonary disease (COPD) (17.4% vs. 17.7%; *p* = 0.935), hypertension (83.9% vs. 86.3%; *p* = 0.300), or dyslipidemia (66.6% vs. 70.8%; *p* = 0.142). In contrast, diabetes mellitus (26.2% vs. 35.8%; *p* < 0.001), chronic kidney disease (3.2% vs. 39.8%; *p* < 0.001) and CAD (60.5% vs. 68.7%; *p* = 0.009) were significantly more common in the higher EASIX group.

**Table 1:** Baseline characteristics according to EASIX cut-off 1.4

	All patients *n* = 1084	EASIX < 1.65 *n* = 654	EASIX ≥ 1.65 *n* = 430	*p*-value
Female	487 (44.9)	332 (50.8)	155 (36.0)	**< 0.001**
Age	82.1 ± 6.1	81.8 ± 6.3	82.3 ± 6.0	0.497
BMI	26.8 ± 5.2	26.6 ± 5.2	26.9 ± 4.9	0.359
COPD	190 (17.5)	114 (17.4)	76 (17.7)	0.935
Hypertension	919 (84.9)	548 (83.9)	371 (86.3)	0.300
Diabetes mellitus	325 (30.0)	171 (26.2)	154 (35.8)	** < 0.001**
Dyslipidemia	740 (68.3)	435 (66.6)	305 (70.8)	0.142
NYHA IV	100 (9.4)	52 (8.0)	48 (11.2)	0.086
Previous MI	148 (13.7)	81 (12.4)	67 (15.6)	0.148
Coronary artery disease	689 (63.6)	396 (60.5)	295 (68.7)	**0.009**
Chronic kidney disease	192 (17.7)	21 (3.2)	171 (39.8)	** < 0.001**
Dialysis	39 (3.6)	1 (0.2)	38 (8.9)	** < 0.001**
eGFR	58.2 ± 13.2	61.0 ± 12.3	43.7 ± 17.2	** < 0.001**
NT-proBNP, pg/ml	2837 (1553/6912)	1564 (628/3494)	3186 (1363/9162)	** < 0.001**
Troponin T, pg/ml	25.3 (18.3/47.8)	21.4 (16.3/33.3)	37.1 (21.0/65.2)	** < 0.001**
Ejection fraction, %	54.9 ± 11.9	55.1 ± 12.3	52.4 ± 13.3	** < 0.001**
AVA, cm^2^	0.73 ± 0.18	0.73 ± 0.17	0.73 ± 0.17	0.208
sPAP, mmHg	37.0 ± 15.4	36.1 ± 16.7	40.3 ± 17.6	** < 0.001**
EASIX	1.91 ± 2.78	1.04 ± 0.34	3.30 ± 4.10	** < 0.001**
EuroSCORE II	5.5 ± 5.4	5.1 ± 4.7	7.5 ± 6.0	** < 0.001**
STS-Score	5.8 ± 3.5	4.5 ± 3.6	6.8 ± 5.9	** < 0.001**
Procedural time, min	72.2 ± 31.7	72.1 ± 31.3	72.4 ± 37.9	0.943

Values are mean (± SD), median (IQR 1/3), or *n*/*N* (%)

*BMI* body mass index, *COPD* chronic obstructive pulmonary disease, *NYHA* New York Heart Association, *MI* myocardial infarction, *eGFR* estimated glomerular filtration rate, *NT-proBNP* n-terminal pro brain natriuretic peptide, *AVA* aortic valve area, *sPAP* systolic pulmonary artery pressure, *STS-Score* Society of Thoracic Surgeons Score. Values in bold indicate statistically significant results (*p* < 0.05)

Dyspnoea at rest with NYHA class IV tends to significance with a borderline p-value (8.0% vs. 11.2%; *p* = 0.086).

Renal and cardiac biomarkers demonstrated notable differences. Patients with an EASIX ≥ 1.65 had significantly lower eGFR values (61.0 ± 12.3 vs. 43.7 ± 17.2; *p* < 0.001) and higher NT-proBNP levels (1564 [628/3494] pg/mL vs. 3186 [1363/9162] pg/mL; *p* < 0.001). Troponin T levels were also elevated in the higher EASIX group (21.4 [16.3/33.3] pg/mL vs. 37.1 [21.0/65.2] pg/mL; *p* < 0.001). Furthermore, left ventricular ejection fraction was reduced (55.1 ± 12.3% vs. 52.4 ± 13.3%; *p* < 0.001), and systolic pulmonary artery pressure was higher (36.1 ± 16.7 mmHg vs. 40.3 ± 17.6 mmHg; *p* < 0.001) in this group.

Procedural factors, including procedural time (72.1 vs. 72.4 min; *p* = 0.943), showed no significant differences. However, patients with an EASIX ≥ 1.65 had a higher EuroSCORE II (5.1 ± 4.7 vs. 7.5 ± 6.0; *p* < 0.001) and STS-Score (4.5 ± 3.6 vs. 6.8 ± 5.9; *p* < 0.001), indicating a higher periprocedural risk.

### Clinical outcomes

**Fig. 1:**
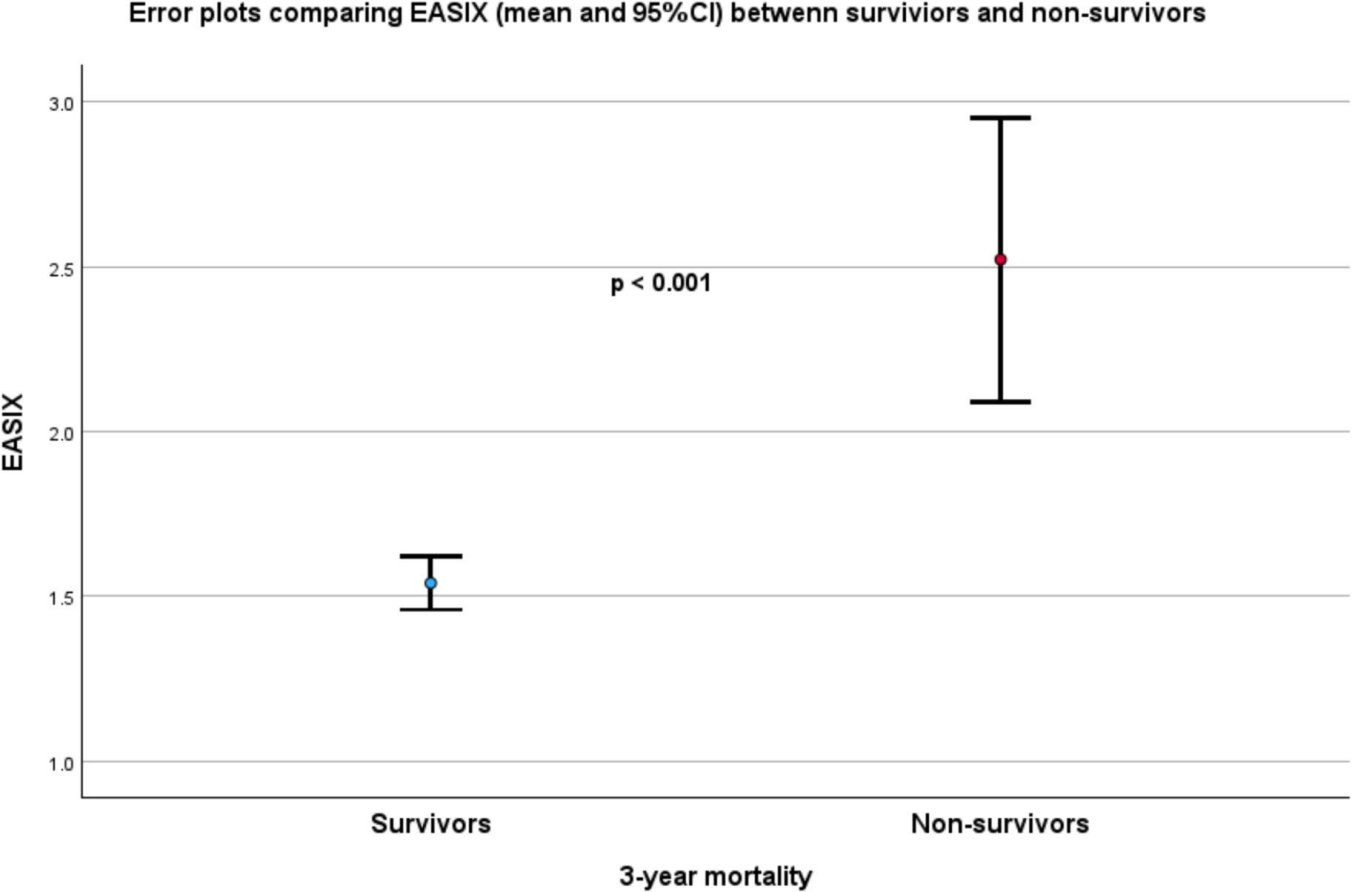
Error bars represent mean and 95% confidence interval values at baseline for survivors and non-survivors based on 3-year mortality. Non-survivors had significantly higher EASIX scores (*p* < 0.001)

Patients who survived after 3 years exhibited significantly lower EASIX levels at baseline than those who did not survive (survival: 1.5 ± 1.0 vs. non-survival: 2.5 ± 4.2; *p* < 0.001), as shown in Fig. 1.

**Fig. 2:**
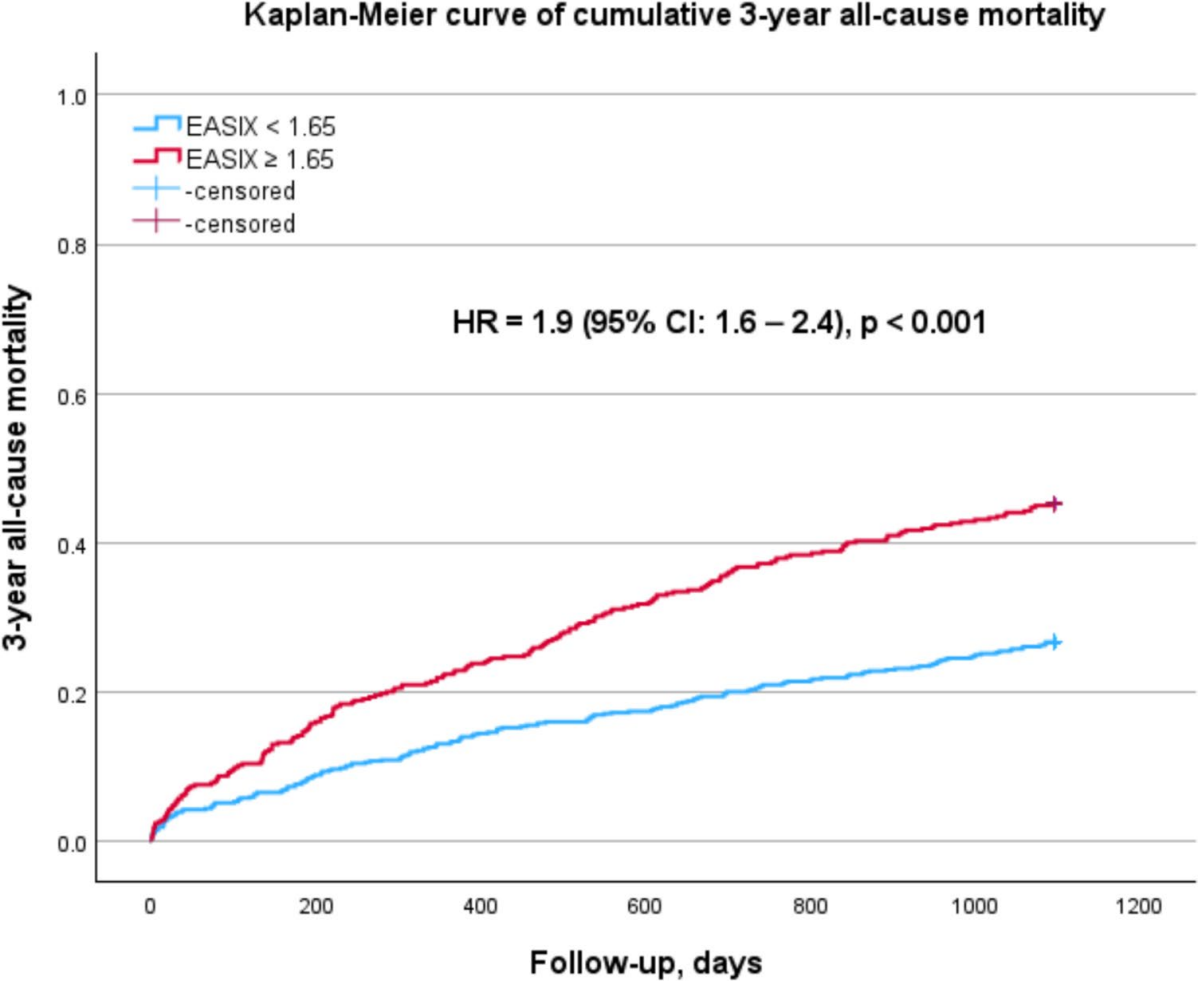
Kaplan–Meier curve of cumulative 3-year all-cause mortality comparing low (< 1.65) and high (≥ 1.65) EASIX groups: significantly higher mortality in the higher EASIX group (HR = 1.9 (95% CI; 1.6–2.4), *p* < 0.001)

Outcome data according to the EASIX revealed statistically significant differences in the 3-year all-cause mortality between the groups. Patients in the lower EASIX group had a significantly lower 3-year mortality rate compared to the high-risk group (27.7% vs. 45.8%; *p* < 0.001) with a hazard ratio (HR) of 1.9 (95% CI: 1.6–2.4; *p* < 0.001) (Fig. 2). As a continuous variable, EASIX was also significantly associated with 3-year all-cause mortality (HR = 1.05, 95% CI: 1.04–1.07; *p* < 0.001). Regarding 30-day all-cause mortality, there were no significant differences between the two groups (low EASIX: 5.0% vs. high EASIX: 6.3%; *p* = 0.416).

In the multivariate Cox regression analysis (Table 2) the EASIX ≥ 1.65 remained independently associated with a higher rate of 3-year all-cause mortality (HR = 1.4, 95% CI: 1.1–1.8; *p* = 0.010) with respect to other confounders. Moreover, diabetes mellitus (HR = 1.5, 95% CI: 1.1–1.9; *p* = 0.007), coronary artery disease (HR = 1.3, 95% CI: 1.0–1.7; *p* = 0.026), and chronic kidney disease (HR = 1.7, 95% CI: 1.2–2.3; *p* < 0.001) were identified as additional significant predictors of increased mortality in multivariate analysis.

**Table 2:** Cox regression analysis for determining predictors of 3-year mortality

Variable	Univariate analysis		Multivariate analysis	
	HR (95% CI)	*p* value	HR (95% CI)	*p* value
EASIX ≥ 1.65	1.9 (1.6–2.4)	**< 0.001**	1.4 (1.1–1.8)	**0.010**
Female sex	0.9 (.07–1.1)	0.409	-	
BMI > 30	1.1 (0.9–1.4)	0.474	-	
NYHA classification	1.6 (1.3–2.0)	**< 0.001**	1.3 (1.0–1.7)	0.082
Chronic kidney disease	2.3 (1.9–2.9)	**< 0.001**	1.7 (1.2–2.3)	**< 0.001**
Diabetes mellitus	1.5 (1.2–1.8)	**< 0.001**	1.5 (1.1–1.9)	**0.007**
Coronary artery disease	1.3 (1.1–1.7)	**0.014**	1.3 (1.0–1.7)	**0.026**
Prior CIED	1.2 (0.9–1.6)	0.325	-	
Ejection fraction < 50	1.4 (1.2–1.8)	**0.002**	1.1 (0.9–1.5)	0.363
Age > 80 years	1.1 (0.9–1.4)	0.224	-	
EuroSCORE II	1.1 (1.0–1.1)	**< 0.001**	1.0 (1.0–1.1)	0.168
STS-Score	1.1 (1.0–1.1)	**< 0.001**	1.0 (1.0–1.0)	0.339

*BMI* body mass index, *NYHA* New York Heart Association, *CIED* cardiac implantable electronic devices, *STS-Score* Society of Thoracic Surgeons Score. ﻿Values in bold indicate statistically significant results (*p* < 0.05)

To evaluate the clinical use of EASIX, we validated two externally defined EASIX cut-offs: EASIX > 2.32, identified as a marker of sepsis-related mortality after alloSCT [16], and EASIX > 1.16, as a predictor of long-term survival in patients undergoing coronary artery bypass grafting (CABG) [17]. We observed a similar hazard ratio, indicating a 1.5- to twofold increased risk of mortality for high EASIX values in our TAVR population (EASIX > 2.32, HR = 1.57; *p* < 0.001) (EASIX > 1.16, HR = 1.93; *p* < 0.001).

### Predictive value of EASIX

**Fig. 3:**
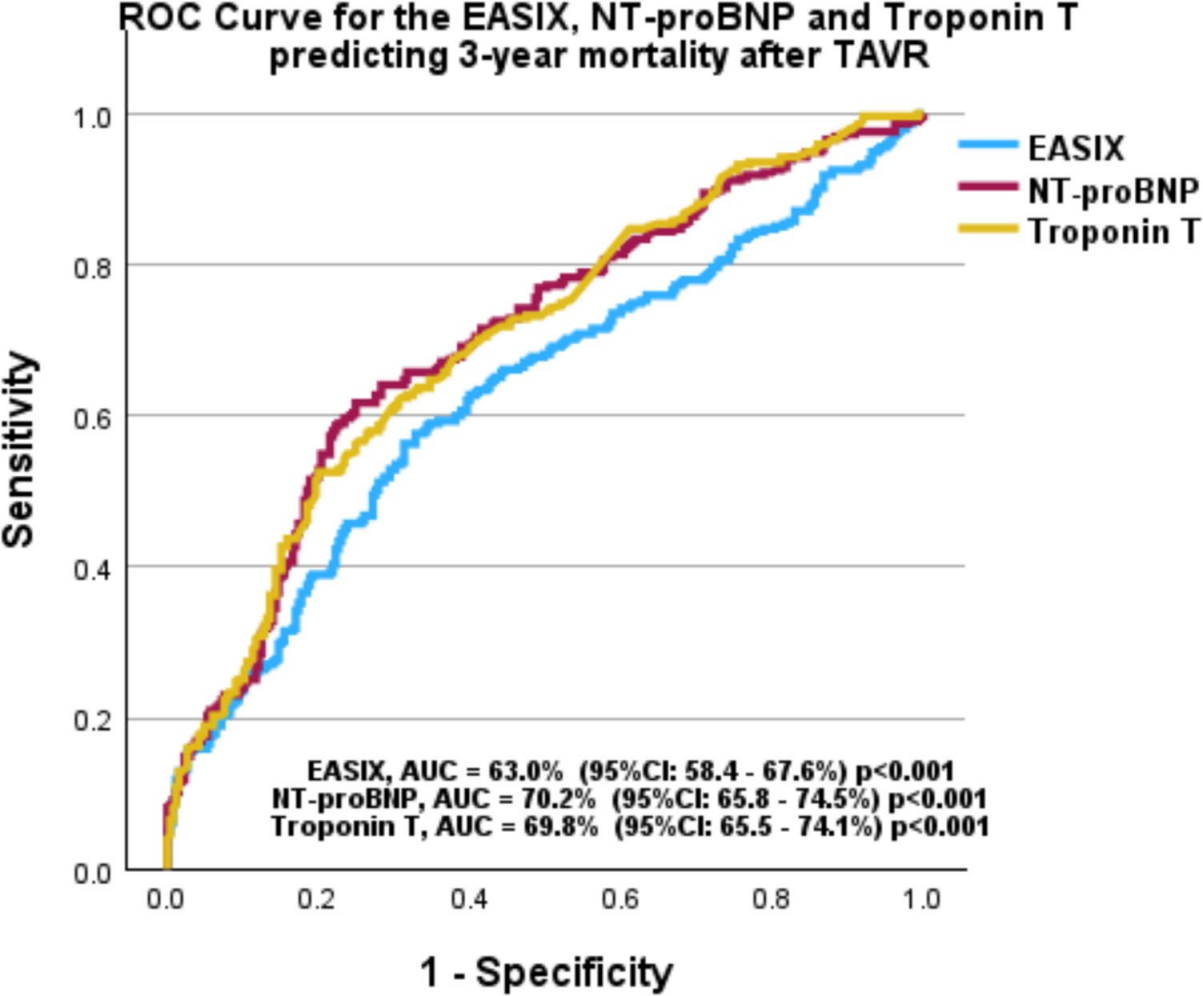
ROC analyses for 3-year all-cause mortality. NT-proBNP and troponin T performed better in predicting 3-year mortality compared to the EASIX. The NT-proBNP achieved an AUC of 70.2% (95% CI 65.8–74.5%, *p* < 0.001), while the troponin T showed an AUC of 69.8% (95% CI 65.5–74.1%, *p* < 0.001). In contrast, the EASIX had a lower AUC of 63.0% (95% CI 58.4–67.6%, *p* < 0.001)

Figure 3 demonstrates the predictive performance of EASIX, troponin T, and NT-proBNP. The ROC analysis showed a good predictive value of EASIX in predicting 3-year mortality with an AUC of 63.0% (95% CI 58.4–67.6%, *p* < 0.001). However, compared to standardized cardiological biomarkers, EASIX lags behind troponin T with an AUC of 69.8% (95% CI 65.5–74.1%, *p* < 0.001) and NT-proBNP with an AUC of 70.2% (95% CI 65.8–74.5%, *p* < 0.001). The DeLong test, used to compare the AUCs, yielded for EASIX vs. troponin T (*z*-value = 1.957, *p* = 0.027) and EASIX vs. NT-proBNP (*Z* = 2.781, *p* = 0.005), indicating that well-established cardiological biomarkers perform significantly better than EASIX in predicting the clinical outcome after TAVR.

Integrating EASIX into a model including troponin and NT-proBNP resulted in a minimal but statistically significant improvement in risk classification. Although the increase in AUC from 75.4% to 76.3% (ΔAUC = 0.9%) did not reach statistical significance (Delong test *Z* =  − 1.196, *p* = 0.232), the categorical Net Reclassification Improvement (NRI) was 3.7% (95% CI: 0.75% to 6.7%, *p* = 0.014), and the continuous NRI was 38.5% (95% CI: 26.5% to 50.5%, *p* < 0.001), indicating a statistically significant improvement in risk classification. Furthermore, the integrated discrimination improvement (IDI) was 3.0% (95% CI: 2.1% to 4.0%, *p* < 0.001), confirming that EASIX enhances risk prediction beyond traditional risk markers.

## Discussion

The present study evaluated for the first time the association of EASIX with mortality in patients with severe aortic stenosis undergoing TAVR. The main results of our study are as follows:

1. Patients with higher EASIX values have a higher cardiovascular risk profile.
2. The EASIX is significantly associated with 3-year mortality after TAVR. Patients with an EASIX ≥ 1.65 had a significantly higher 3-year mortality compared to those with an EASIX < 1.65.
3. After adjusting for relevant confounders, EASIX ≥ 1.65 remained an independent predictor of higher 3-year all-cause mortality.
4. The AUC values for troponin T and NT-proBNP demonstrated superior predictive performance for 3-year mortality compared to EASIX.

EASIX comprises biomarkers linked to cellular damage, renal function, and coagulation – key factors associated with endothelial dysfunction [18]. A high EASIX score was found to correlate with increased endothelial activation markers, including tumor inhibitory factor-2, angiopoietin-2, soluble thrombomodulin, interleukin-18, chemokine-X-C-ligand 8, chemokine-X-C-ligand 9, insulin-like-growth-factor-1, soluble thrombomodulin, and recently sCD141 [7, 12, 19].

Endothelial dysfunction is a major contributor to numerous pathological conditions, including thrombosis, cardiovascular disease, diabetes, and cancer, as it is influenced by vascular inflammation, reactive oxygen species production, and endothelial damage [20, 21]. This suggests that persistent endothelial dysfunction could play a central role in cardiovascular morbidity by promoting atherosclerosis, inflammation, and thrombosis [22, 23], which may explain the association between higher EASIX values and increased cardiac toxicity, including heart failure and arrhythmias, in patients undergoing hematopoietic cell transplantation [24]. Our study results align with this literature, showing that patients with higher EASIX values are associated with an unfavorable cardiovascular profile — characterized by a higher prevalence of diabetes mellitus, CAD, and chronic kidney disease, as well as elevated cardiac and renal biomarkers and reduced left ventricular ejection fraction.

It is known that human aortic stenotic valve-derived extracellular vesicles induce endothelial dysfunction and thrombogenicity [25]. Furthermore, AS increases post-valvular swirling blood flow in the central ascending aorta, triggering red blood cell fragmentation with the accumulation of hemoglobin in the plasma, leading to systemic endothelial dysfunction [26]. EASIX, as a marker of endothelial dysfunction, has been identified as a significant prognostic factor for mortality in various conditions, including haemato-oncological diseases, such as multiple myeloma [27] and diffuse large B-cell lymphoma [28], as well as in acute inflammatory diseases like acute pancreatitis [29] and sepsis [10], and more recently, in acute myocardial infarction [14]. The three individual components of the EASIX score—LDH [30], creatinine [31], and platelets [32]—are well-established predictors of cardiovascular mortality, and Fink et al. suggested EASIX as a potential prognostic marker of survival in patients with CAD [13]. In our study of AS patients undergoing TAVR, we identified a significant association between the EASIX score and 3-year mortality. Notably, EASIX emerged as a stable and independent predictor of long-term mortality following TAVR, even after adjusting for potential confounders such as sex, COPD, hypertension, diabetes mellitus, CAD, and dyslipidemia.

NT-proBNP and troponin T have been validated as robust predictors of mortality and adverse events in patients with aortic stenosis undergoing TAVR [33, 34]. However, a direct comparison between the EASIX score and these established cardiovascular biomarkers is still lacking. In our analysis, NT-proBNP and troponin T demonstrated superior predictive value for long-term outcomes compared to EASIX. This discrepancy may be explained by the nonspecific nature of the EASIX score and its components. Furthermore, comorbid conditions such as chronic kidney disease or liver disease can artificially elevate EASIX scores, potentially reducing its accuracy in predicting mortality.

Accurate individual risk stratification is critical for guiding clinical decision-making in patients undergoing TAVR. Consequently, a comprehensive approach to risk assessment could incorporate established cardiac biomarkers (e.g., NT-proBNP and troponin) alongside the EASIX score. This integrated strategy would provide a more nuanced assessment of patient risk and enhance predictive accuracy. Such considerations could inform the development of more personalized risk models to further refine prognostication and improve patient outcomes.

## Limitations

The main limitation of this study is its retrospective, single-center design, which may have introduced selection bias. The follow-up duration of three years, while providing useful insights into medium-term outcomes, does not capture long-term survival, and a longer follow-up would be beneficial for understanding the enduring predictive value of EASIX. Furthermore, the study lacks data on the longitudinal course of EASIX after TAVR, which could provide valuable information on whether an improvement occurred and whether such an improvement has an impact on mortality. Due to the retrospective design, certain potentially relevant clinical and echocardiographic parameters could not be systematically analyzed.

## Conclusion

EASIX is a significant predictor of 3-year all-cause mortality in patients undergoing TAVR. An EASIX above 1.65 is independently associated with increased mortality risk, even after adjusting for established clinical risk factors, emphasizing its potential as a useful biomarker in predicting long-term outcomes following TAVR. However, its predictive ability was still inferior to well-established cardiovascular biomarkers such as NT-proBNP and troponin T. Integrating EASIX with traditional cardiac biomarkers may enhance risk stratification in TAVR patients and improve personalized care.

## Abbreviations

AS: Aortic valve stenosis
TAVR: Transcatheter aortic valve replacement
EASIX: Endothelial Activation and Stress Index
TAM: Transplant-associated thrombotic microangiopathy
alloSCT: Allogeneic stem cell transplantation
GVHD: Graft-versus-host disease
CAD: Coronary artery disease
eGFR: Estimated glomerular filtration rate
NT-proBNP: N-terminal pro b-type natriuretic peptide
COPD: Chronic obstructive pulmonary disease
BMI: Body mass index
NYHA: New York Heart Association
eGFR: Estimated glomerular filtration rate
EUROSCORE II: European System for Cardiac Operative Risk Evaluation II
STS PROM: Society of Thoracic Surgeons Predicted Risk of Mortality
